# Correction: Peptide epitope-imprinted polymer microarrays for selective protein recognition. Application for SARS-CoV-2 RBD protein

**DOI:** 10.1039/d3sc90170j

**Published:** 2023-09-05

**Authors:** Zsófia Bognár, Eszter Supala, Aysu Yarman, Xiaorong Zhang, Frank F. Bier, Frieder W. Scheller, Róbert E. Gyurcsányi

**Affiliations:** a BME “Lendület” Chemical Nanosensors Research Group, Department of Inorganic and Analytical Chemistry, Budapest University of Technology and Economics Szt. Gellért tér 4 1111 Budapest Hungary gyurcsanyi.robert@vbk.bme.hu; b Institute of Biochemistry and Biology, University of Potsdam Karl-Liebknecht-Str. 24-25 14476 Potsdam OT Golm Germany

## Abstract

Correction for ‘Peptide epitope-imprinted polymer microarrays for selective protein recognition. Application for SARS-CoV-2 RBD protein’ by Zsófia Bognár *et al.*, *Chem. Sci.*, 2022, **13**, 1263–1269, https://doi.org/10.1039/D1SC04502D.

The authors regret that the funding details were incomplete in the Acknowledgements section of the original article. In addition to the original acknowledgements the following text should have been included:

The research reported in this paper is part of Project BME-EGA-02, implemented with support provided by the Ministry of Innovation and Technology of Hungary from the National Research, Development and Innovation Fund, financed under the TKP2021 funding scheme.

The Royal Society of Chemistry apologises for these errors and any consequent inconvenience to authors and readers.

## Supplementary Material

